# Functional Assessment of Pharmacological Telomerase Activators in Human T cells

**DOI:** 10.3390/cells2010057

**Published:** 2013-01-14

**Authors:** Brenda Molgora, Riley Bateman, Greg Sweeney, Danielle Finger, Taylor Dimler, Rita B. Effros, Hector F. Valenzuela

**Affiliations:** 1 Department of Biology, Whittier College,13406 Philadelphia Street, P.O. Box 634, Whittier 90608, CA, USA; E-Mails: bmolgora@poets.whittier.edu (B.M.); rbateman@poets.whittier.edu (R.B.); gsweeney@poets.whittier.edu (G.S.); dfinger@poets.whittier.edu (D.F.); mdimler@poets.whittier.edu (T.D.); 2 Department of Pathology and Lab Medicine, David Geffen School of Medicine at UCLA, 10833 Le Conte Avenue, Los Angeles 90095-1732, CA, USA; E-Mail: Reffros@mednet.ucla.edu

**Keywords:** TA-65, telomerase activators, cellular senescence

## Abstract

Telomeres are structures at the ends of chromosomes that shorten during cell division and eventually signal an irreversible state of growth arrest known as cellular senescence. To delay this cellular aging, human T cells, which are critical in the immune control over infections and cancer, activate the enzyme telomerase, which binds and extends the telomeres. Several different extracts from the *Astragalus membranaceus* root have been documented to activate telomerase activity in human T cells. The objective of this research was to compare two extracts from *Astragalus membranaceus*, TA-65 and HTA, for their effects on both telomerase and proliferative activity of human CD4 and CD8 T cells. Our results demonstrate that, TA-65 increased telomerase activity significantly (1.3 to 3.3-fold relative to controls) in T cell cultures from six donors tested, whereas HTA only increased telomerase levels in two out of six donors. We also demonstrate that TA-65 activates telomerase by a MAPK- specific pathway. Finally, we determine that during a three-day culture period, only the T cells treated with the TA-65 extract showed a statistically significant increase in proliferative activity. Our results underscore the importance of comparing multiple telomerase activators within the same experiment, and of including functional assays in addition to measuring telomerase activity.

## 1. Introduction

Telomeres are regions at the ends of chromosomes characterized by a repeating TTAGGG sequence. Furthermore, telomeres have specific associated proteins, collectively referred to as shelterin proteins, that help stabilizes this chromosome region, thereby playing a key role in chromosome stability, gene regulation, cancer and cellular senescence [1,2]. During DNA replication, DNA is synthesized by a continuous leading strand and by a discontinuous lagging strand, which are primed by an RNA polymerase. However, because of steric hindrance between the DNA polymerase and the required RNA priming at the ends of chromosomes, the lagging strand cannot be completely synthesized. Hence, with each cell division, the DNA length shortens at the ends within the telomere region. As telomeres shorten, the chromosome ends become reactive, leading to chromosomal fusions, resulting in DNA molecules with two centromeres, referred to as a dicentric chromosome [3]. During cell division, dicentric chromosomes can be pulled apart in opposite directions, leading to breaks in the chromosomes, thus repeating the cycle in a process known as breakage-fusion-bridge cycles [4]. The telomere region, therefore, acts to prevent these fusions by capping the ends of the chromosomes and maintaining DNA integrity. 

Telomerase is a reverse transcriptase ribonucleoprotein enzyme that extends the leading strand of DNA by adding TTAGGG repeats to the end of the telomere. The dynamic maintenance of telomere length by telomerase has been found to strongly correlate with both the limited proliferative lifespan of normal cells and the immortality of cancer cells. Low to undetectable levels of telomerase activity are present in most somatic cells, leading to telomere shortening with every cell division [5,6]. Without telomere length maintenance, DNA will be damaged or deleted in cells that undergo extensive proliferation, whereas robust telomerase activity in cancer cells allows for extensive proliferative-potential.

Some human cells, such as epithelial cells in the skin and cells of the immune system require many cell divisions in order to carry out their physiological functions. These cells show detectible, albeit low, telomerase activity. However, when T cells are stimulated, either through their T cell antigen receptor or with mitogens, they are able to upregulate telomerase activity that is in the same range as that observed in tumor cells [7]. Activation of robust telomerase within T cells is critical, since low telomerase activity has been shown to lead to a premature decline of the immune system. T cell induction of telomerase is, nevertheless, a transient event, and decreases significantly with increasing rounds of cell division; thus, T cell’s telomeres ultimately shorten to a critical length and signal senescence [8,9]. As further evidence of the critical role of telomerase in aging, a number of *in vitro* and *in vivo* studies have shown that activating telomerase can not only delay aging but also reverse age tissue degeneration [10,11]. 

Since both normal and abnormal (including cancer) cells must maintain genomic integrity, telomere and telomerase research has been a key area of investigation in such diverse fields as aging, cancer and pathogen-driven chronic degenerative diseases [12]. Moreover, given the demographic shift and the ever-growing aging population, regenerative medicine is also focused on strategies to maintain telomere length. Gene therapy with hTERT, the catalytic component of telomerase, though successful in cell culture, is not a practical medical intervention. An attractive alternative would be a chemical telomerase activator, which would allow for a more precise control over the dose and timing. Several extracts from the *Astragalus membranaceus* root are being studied as possible telomerase activators [11,13,14]. The goal of the present study was to compare two natural extracts from the *Astragalus membranaceus* root, TA-65 and HTA, for their capacity to enhance telomerase activity and proliferation in human CD4 and CD8 T cells. This preliminary study highlights the importance of comparative assessments of new activators of telomerase within single experiments in evaluating them as treatments for age-associated pathologies or for immuno-compromising chronic diseases.

## 2. Results

### 2.1. TA-65 but Not HTA Increased Telomerase Activity in All Donors’ T Cells during Primary and Secondary Stimulations

**Figure 1:**
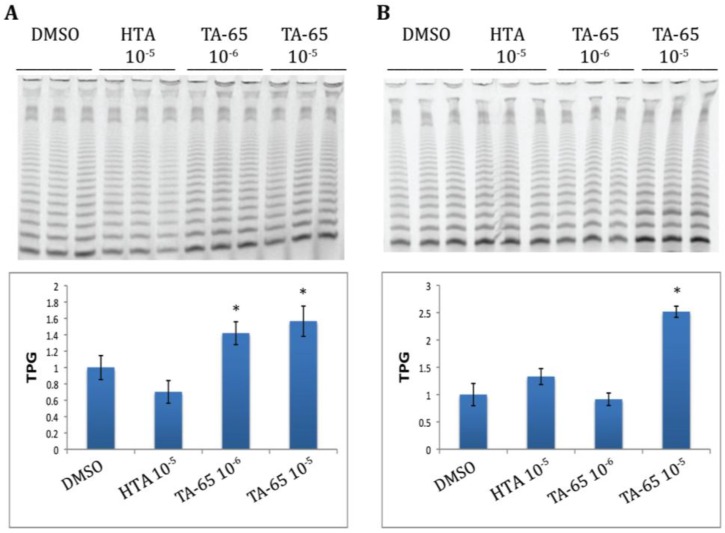
TA-65 and HTA treatment of CD4 T cells increases telomerase activity in response to primary (**A**) and secondary (**B**) cell stimulations. This figure shows representative results for cultures of purified CD4 T cells from a single donor that were exposed to either TA-65, HTA or DMSO (triplicate cultures for each condition). Top portion of Figure 1A,B illustrate the band products of telomerase activity indicated as TPG (TPG is total product generate, a measure of telomerase activity). Dark bands indicate high telomerase activity. The bottom portions of panels A and B represent quantization bar graph values for the enzyme activity. The average DMSO TPG was used to normalize telomerase activity between treatments. The total dilution of compounds in grams/mL is indicated above the lanes and below the bar graphs. Statistical significance was determined by student paired t test (*p* < 0.05).

**Figure 2:**
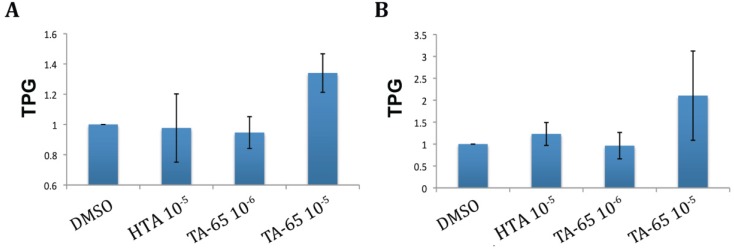
Average telomerase activity for a primary stimulation for (**A**) CD4 (n = 6) and (**B**) CD8 (n = 6) T cells. TPG is total product generated for telomerase activity. DMSO treated samples were used to normalized the TPG between the donors, which allow direct donor’s variability comparisons.

Cultures were established from purified CD4 and CD8 T cells from six healthy donors. The cells were treated with TA-65, HTA, or DMSO (diluent control) and samples were taken to measure telomerase activity 72 h after primary stimulation and the process repeated after 18–21 days for a secondary stimulation. Representative examples of CD4 T cell telomerase activity from one of the donor’s cultures following both primary and secondary stimulations are illustrated in Figure 1A,B, respectively. The top panels represent actual bands obtained in the TRAP gel, and the corresponding quantifications are graphed on the bottom. Our results show that during a primary stimulation, TA-65 at both 10^−5^ and 10^−6^ gm/mL dilution increased telomerase activity on average 1.57 to 1.42 fold, respectively, when compared to the DMSO control, (Figure 1A). In a subsequent stimulation of the same cells (at the same TA-65 concentration) 19 days after the initial stimulation, telomerase activity was 2.51 fold greater than the control in the TA-65 treated cultures at 10^−5^ gm/mL dilution but at 10^−6^ gm/mL dilution the telomerase activity was at the same level as the control (Figure 1B). By contrast, HTA, had no effect on the telomerase activity following first stimulation, and caused only a modest 1.3-fold increase following second stimulation, which did not reach statistical significance (Figure 1A,B). We also observed that in some cases treating with the compounds appeared to reduce the telomerase activity for some donors when compared to the DMSO controls. However, this apparent decrease in telomerase activity, did not reach statistical significance. A compilation of the mean telomerase activity values (normalized to the means of their respective DMSO controls) for six donors is summarized in Figure 2, Panel 2 A illustrates average telomerase activity for treated CD4 T cells, and Figure 2B shows averages for treated CD8 T cells. Although not discernible in Figure 2A,B, there was a slight trend in upregulation of telomerase activity in the HTA (10^−6^ gm/mL) treated cultures for some donors during a second stimulation, but only in two out of six donors did this reach statistical significance. When results of all six cultures were evaluated, the HTA-mediated effect failed to reach statistical significance. However, in all cultures treated with the TA-65 compound (dilution 10^−5^ gm/mL), the increase in telomerase activity was statistically significant.

### 2.2. MAPK Specific Inhibitor Blocks TA-65 Induced Telomerase Activity

**Figure 3:**
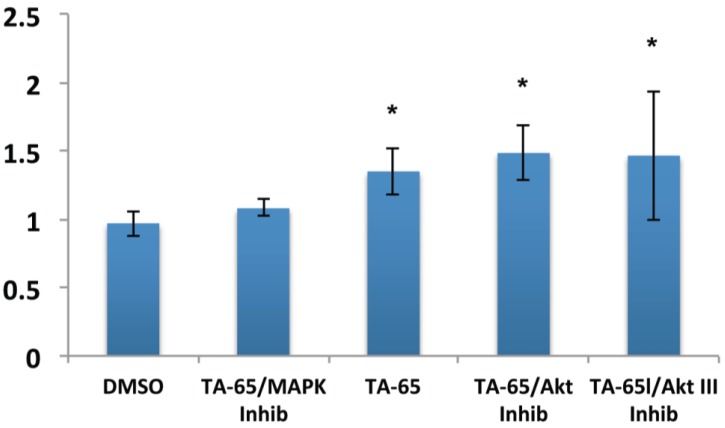
TA-65 activates telomerase via MAPK pathway. CD8 T cells stimulated for a second time and treated with MAPK inhibitor (as described in materials and methods) blocked the increase telomerase activity from TA-65, but no significant inhibition was observed using AKT inhibitors. Statistical significance was determined by student paired t-test (*p* < 0.05).

Previous telomerase activators tested in earlier studies in our lab were linked to MAPK/ERK pathway [13]. To elucidate whether TA-65 also activates telomerase through the MAPK pathway, we compared the ability of MAPK and AKT inhibitors to block TA-65 induced telomerase activity (Figure 3). Our results show that TA-65 likely uses the MAPK pathway to activate telomerase, based on the reduction of the telomerase activity in the presence of the MAPK inhibitor. This result was observed in both CD4 and CD8 T cells but most pronounced in CD8 T cells that have been stimulated for a second time (Figure 3). The AKT pathway similar to our previous results with other telomerase activators did not seem to have a significant effect on telomerase activity on either CD4 or CD8 T lymphocytes. 

### 2.3. Telomerase Activator TA-65 but not HTA Increases T Cells Proliferation

**Figure 4:**
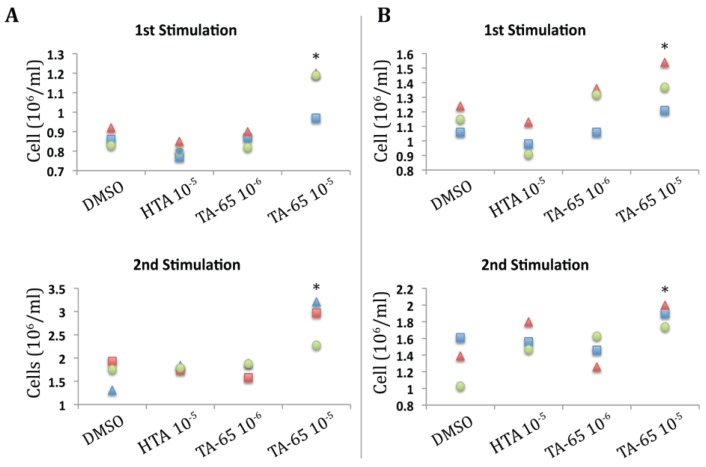
Proliferation effects of TA-65 and HTA: Cell counts were determined on day 3 after stimulation. A, represents results for CD4 T cells and B represents results for CD8 T cells (concentrations are 10^6^/mL). A and B top, represents actual cell concentration after first T cell stimulation, A and B bottom represents total cell concentration after second stimulation. Statistical significance was determined by student paired t test (*p* < 0.05).

Our previous work on the TAT-2 telomerase activator had shown that the enhanced telomerase activity was associated with increased long-term proliferative capacity of the T cells [13]. For this study we decided to test for the earliest significant telomerase activity. Here, we show that exposure to TA-65 at a dilution 10^−5^ gm/mL for even as short a period as 3 days is able to induce significant increases in proliferation. Indeed, both CD4 T cells (Figure 4A) and CD8 T cells (Figure 4B) experienced greater degrees of proliferation in the presence of TA-65 following both the first and second rounds of stimulation. This effect would be particularly critical during acute viral infection, since rapid early vigorous proliferation is essential for effective immune control over the infection. By contrast, exposure to HTA failed to induce increased proliferative activity during the 3-day culture period after either first or second stimulations.

## 3. Discussion

In the current study we have characterized the effects of two extracts of *Astragalus membranaceus*, TA-65 and HTA, for their ability to induce telomerase activity and cell proliferation on both CD4 and CD8 T cells. We show that TA-65 increased CD4 and CD8 T cells telomerase activity by 1.3-3.3-fold over the control treatment (*p* < 0.05) in six out of six donor cultures, but HTA treatment caused mild telomerase increases in only 2 of the 6 donor cultures. The TA-65 telomerase enhancement was in the same range as that documented in our previous work with the TAT2 telomerase activator on CD8 T cells from persons infected with HIV [13]. In that study we observed telomerase enhancement of 1.5 to 2.5 fold increase over control, a change that was associated with a significant increase in the ability of the T cells to reduce HIV virus production when co-cultured with infected cells. Thus, our data suggest that TA-65 may be useful in treating both HIV disease and other clinical situations requiring enhanced T cell telomerase activity. Our study also demonstrated that the TA-65 induction of telomerase activity was most likely mediated via the MAPK, rather than the AKT pathway due to the higher sensitivity to MAPK inhibitors, consistent with results from TAT2 [13]. Importantly, our experiments highlighted donor variability with respect to the cell proliferation effects of both compounds. Our results show that TA-65 increased proliferative capacity of T cells, but there was variability among the donors; the 10^−5^ gm/mL dilution worked on all cultures, whereas the 10^−6^ gm/mL dilution only worked with some donors. By contrast, no statistical difference in cell growth over the control cultures was observed for HTA for all concentrations tested. 

Our results also accord with several *in vivo* studies done on both rodents and humans using telomerase activators [11,14]. In one study, mice treated with TA-65 showed similar increased telomerase enzymatic activity, and increases in health span indicators [11]. This study showed that the effects of the TA-65 included not only rescuing critically short telomeres, but also improving health parameters such as glucose tolerance, osteoporosis and skin fitness [11]. The report describing initial research in humans treated with TA-65 showed enhancement of certain immune parameters that had previously been associated with beneficial health effects [14]. The most important T lymphocyte-related observation made in that study was the decline in the proportion of T cells with a senescent phenotype (*i.e*., CD8+CD28-), thus restoring the peripheral blood T cell composition to a more youthful profile. 

The ability to regulate telomerase activity is viewed as very therapeutically important in the fields of aging and cancer. Studies on the dynamic link between telomeres, cancer and aging in the 1990’s dramatically increased interest in the field of telomere and telomerase biology. Early seminal papers described the discovery that telomere length functioned as a cellular mitotic clock for aging [15,16]. Other key studies that further elucidated the relationship between cellular senescence and cancer involved genetic modifications of telomerase expression, leading to immortalization of primary epithelial tissue without inducing cancer [17]. Nevertheless, conflicting results have been reported with regard to telomerase expression and induction of proliferation in different tissues. Telomerase alone cannot induce immortalization in all tissues, suggesting tissue-specific regulatory mechanisms to control telomerase activity. For example, primary human T cells regulate their telomerase activity more stringently than primary epithelial cells. Our own previous studies demonstrated that telomerase can be both upregulated and downregulated in normal T cells upon cell activation, depending on certain co-stimulatory signals [8,18], and that gene transduction with the telomerase catalytic protein (hTERT) lead to significantly enhanced proliferation and anti-viral function, with no sign of transformation [12]. Yet there still remains some controversy due to conflicting reports on whether sustained expression of ectopic hTERT alone is sufficient to immortalize CD8 T cells [19,20]. The ability to strictly control telomerase activity in T cells is critically important in developing treatments for aging, for example, since impaired signals can lead to immunodeficiencies, while unrestrained signals could theoretically lead to autoimmunity and leukemia. Importantly, none of the studies to date have shown any negative effects of telomerase enhancement on T cells regulation. Furthermore, in mice it has been demonstrated that telomere rejuvenation of the tissues and organs may lead to extension of lifespan [10]. Nonetheless, gene therapy has many drawbacks, and nongenetic activation of telomerase via natural compounds may be a more effective and less invasive approach.

## 4. Experimental Section

### 4.1. Formulation and Treatments of TA-65 and HTA

TA-65 and HTA were obtained from RevGenetics. TA-65, which is a single chemical entity, is isolated by a proprietary purification process from the extract of the root of *Astragalus membranaceus* (licensed exclusively to TA Sciences) [11,14]. HTA is also an extract of the root of *Astragalus membranaceus* but is not a single purified compound entity. For use in the T cell cultures, the equivalent of 0.05 gm of each compound was dissolved in 10 ml of DMSO to achieve stock concentrations of 5 × 10^−3^ gm/mL. Serial dilutions ranging between 10^−3^, 10^−4^, 10^−5^ and 10^−6^ gm/mL were tested for each compound on the T cell cultures. The total volume of the compounds added for each concentration into the experimental cultures represented 0.1% of the final volume. 

### 4.2. Cell Treatment and Stimulation

Human peripheral blood samples were acquired after informed consent, and in accordance with both the Human Subject committee at Whittier College and the University of California-Los Angeles Institutional Review Board. T cells from PBMC were isolated and maintained as described previously [13]. Briefly, peripheral blood mononuclear cells (PBMC) where separated from blood samples by Ficoll-Hypaque gradient centrifugation. CD4 and CD8 T cells were purified from the PBMC by negative selection, using a CD4 and CD8 T cell isolation kit (Miltenyi Biotec) according to the manufacturer’s instructions. The T cells were stimulated with CD2/CD3/CD28 antibody-coated beads (Milteny) at a 0.5:1 bead to cell ratio and cultured in RPMI 1640 media supplemented with 10% FBS, 2 mM glutamine, 1 mM HEPES, 50 IU/mL penicillin/streptomycin and recombinant IL-2 (50 U/mL). T cells were then treated with the compounds and retreated every 48 h as described. All treatments for each condition were performed in triplicate wells. Cells were counted by trypan blue exclusion every 48–72 h, and were sub-cultured to 5 × 10^5^ cells/mL when cell concentration exceeded 1 × 10^6^ cells/mL. T cell cultures were re-stimulated for a second time (secondary stimulation) approximately 18–21 days after the 1st stimulation. 

### 4.3. Cell Signaling Inhibitors

Cell signaling pathway inhibition was done as described [13]. T cells were stimulated for a second time with CD2/CD3/CD28 antibody-coated beads and IL-2 for seven days. Cells were then washed and treated for 2 h with RPMI media and with one of the following cell signaling inhibitors: MAPK inhibitor PD98059 (Calbiochem no. 51300), AKT I inhibitor (Calbiochem no. 124009) and AKT III (Calbiochem no. 124005). Cells were then washed with RPMI media and treated with TA-65 and DMSO as described above. Cell samples were taken 16 h later for telomerase analysis.

### 4.4. Telomerase Activity Measurements

T cell samples (1 × 10^6^/mL) collected 72 h after either the first or second round of stimulation were washed in PBS buffer and stored at −80 °C. Telomerase activity was measured using the telomeric repeat amplification protocol (TRAP), as described in Parish et al [18]. The amplified TRAP reactions were run in triplicates for each condition, and reaction products were separated in a 12.5% polyacrylamide gel, with the resulting bands visualized and analyzed using Storm 860 software (GE Healthcare). Statistical analysis was done using a two tail Student’s t test and significance established with a p value of < 0.05.

## 5. Conclusions

*Astragalus membranaceus* root extracts have traditionally been used for centuries as Chinese herbal teas for their health-promoting properties. Although most claims are anecdotal in nature, some have been substantiated in recent scientific studies [21,22,23]. However, no studies have directly compared the different compounds from the *Astragalus membranaceus* roots extracts (*i.e*., TA-65, TAT2, HTA and Astragaloside IV). It stands to reason that not all *Astragalus membranaceus* roots extracts can stimulate telomerase activity to the same degree, hence our interest in the current study. Although our results indicated that the HTA in cultures stimulated telomerase activity in T cells in only two out of six donors, the small sample size may be a weakness to this study. Further studies should include a larger sample size, which could help confirm or change our observations. Nevertheless, we feel that this preliminary study begins to lay the foundation for, not only comparing different *Astragalus membranaceus* root extracts, but also other natural telomerase activators. 
